# Understanding Medical Students’ Decision Making in Relation to Seeking Help for Mental Health Problems

**DOI:** 10.1007/s40596-025-02212-9

**Published:** 2025-09-22

**Authors:** Amy E. Manley, Paul Moran, Jelena Savović, Lucy Biddle

**Affiliations:** 1 Population Health Sciences, Bristol Medical School, University of Bristol, Bristol, UK; 2 NIHR Applied Research Collaboration West (ARC West) at University Hospitals Bristol and Weston NHS Foundation Trust, Bristol, UK

**Keywords:** Medical students, Mental health, Help-seeking, Stigma, Stress

## Abstract

**Objective:**

This qualitative study explored medical students’ decision making in relation to seeking help for mental health problems.

**Methods:**

Fourteen medical students participated in a set of three semi-structured interviews. Interviews explored students' experiences of distress and professional help-seeking for mental health problems. Data were analyzed using thematic analysis.

**Results:**

All students experienced “stress” and seven had previously sought help. In deciding whether to seek help, students considered the meaning of difficult feelings, often attributing them to a stressful career. They considered mental health problems to be a sign of “weakness” which threatened their valued professional identity as a “strong” caregiver. Students questioned the acceptability of seeking help for mental health symptoms and believed they should know better than to seek help inappropriately. Concerns about confidentiality and career implications also led to reluctance to disclose symptoms, particularly thoughts of self harm. Busy timetables and distant clerkships imposed additional practical barriers to accessing support.

**Conclusions:**

The study highlights the complex interplay of individual, social, and cultural factors influencing medical students' help-seeking decisions. The findings resonate with sociological models of help-seeking, emphasizing the role of identity and perceived norms in shaping decisions. Medical educators should emphasize the importance of mental health in clinical as well as pastoral settings, and challenge unhelpful professional norms and stigma towards mental illness. Greater recognition of students' tendency to downplay suicidal thoughts may aid professionals in providing appropriate and timely support.

Mental health problems are common in medical students, with over a quarter reporting depressive symptoms [1, 2]. Such difficulties can adversely affect academic performance and patient care [3]. However a gap exists between the high prevalence of mental health symptoms among medical students and doctors, and the uptake of formal support, with a meta-analysis revealing as few as 15.7% of depressed medical students sought treatment internationally [2], rising to between 22 and 53% of depressed US medical students [4, 5]. Studies have suggested barriers to help seeking include fears about stigma, confidentiality, career repercussions, and lack of time within demanding schedules [4–6]. However interventions to address such factors have only limited, temporary effects on attitudes to seeking professional help [7, 8]. A better understanding of help-seeking decision-making in this population could inform the development of interventions to promote timely help-seeking for mental health problems.

Psychological and sociological models have been developed to explain help-seeking behavior in the general population. Psychological models focus on cognition and the individual factors that influence help-seeking. For example, the Health Belief model [9] postulates that individual perceptions of illness severity, their susceptibility, and the benefits and costs of seeking help determine subsequent behavior. Empirical evidence suggests this model predicts around half of the variance in treatment seeking for common mental disorders in the general population [10]. The postulation that help-seeking decisions are heavily reliant on understanding illness and treatment [11] might generate the expectation that medical students would seek help more frequently, given their enhanced knowledge about mental health problems [12]. Yet current evidence suggests that medical students show average levels of help-seeking when compared with students from other academic programs [13–16].

Sociological models emphasize the role of social and cultural factors in shaping help-seeking behavior. These highlight the importance of the interpretation of symptoms and “meaning” people attach to help-seeking [17, 18], the consultation with friends and family to reach an informal diagnosis [19] and social norms including stigma [20], in influencing help-seeking decisions. Such models view help-seeking as a dynamic series of smaller decisions, with outcomes iteratively influencing help-seeking behavior [21]. Previous studies have recognized the influence of stigma on help-seeking across student groups and in the general population [6, 22–24], yet stigma towards mental illness has been shown to be no greater in medical students than other student groups [25]. Furthermore, few existing studies consider how broader sociological influences at medical school may influence help-seeking, and why such influences arise. This study seeks to better understand the process through which medical students decide whether or not to seek help for mental health problems from healthcare and university services.

## Methods

### Study Design

A qualitative study involving individual in-depth interviews with medical students was undertaken. An interpretivist approach facilitated understanding of the diverse influences on the help-seeking decision making process, rather than seeking a ‘unifying truth’. The study was part of a larger study investigating student experiences during a psychiatry clerkship [26]. Ethical approval was obtained from the Faculty of Health Science Research Ethics Committee at the University of Bristol (39101).

### Population and Sampling

One-hundred and six fourth-year medical students were invited by email to participate in the research. Students were due to commence consecutive psychiatry clerkships, which represented their first clinical exposure to the specialty. Twenty-four students (23%) responded, from whom fifteen participants were purposively selected to maximize variability in the sample according to gender, location of schooling (within / outside the UK), openness to pursuing a psychiatric career and clerkship location. Purposive sampling aimed to capture a breadth of perspectives, rather than achieving proportional representation of cohort demographics. Recruitment was not based on symptomatology or prior help-seeking behavior. All participants gave informed consent to participation. After fifteen students had been recruited, the collected interview data was deemed to have sufficient information power to meet the aims of the broader study and recruitment ceased. One student withdrew following the first interview, and their data were not used. The use of relevant probes encouraged in-depth discussion about help-seeking decisions, resulting in high quality dialogue relevant to the study aim. This, as well as a relatively homogenous population (all medical students), reduced sample size requirements, whilst an inductive approach increased them [27]. Therefore collected data from the fourteen remaining students continued to yield good information power for the aim of this study.

### Data Collection

Each student was interviewed by AM (a psychiatrist) at three time points over a 10-week period, enabling the building of trust with the interviewer, thus supporting disclosure about any experiences of mental health difficulties and enhancing data quality. At the time of the study, the interviewer had no role in clerkship organization or bearing on students’ academic progress. Semi-structured interviews were steered by a topic guide, using open-ended questions to explore students’ experiences of symptoms and mental health help-seeking, both personally and amongst their friends and family. As the interviews progressed, refinements were made to the topic guide to ensure iterative exploration of emerging themes. Interviews were approximately one hour long, audio-recorded, and transcribed verbatim. Participants' travel expenses were reimbursed.

### Data Analysis

Each participant’s set of interviews were collated and analyzed together. Thematic analysis followed a method adapted from Braun and Clarke [28]. Transcripts from 3 students were independently double coded by a second researcher (LB) to offer a non-clinical perspective on the data. Codes were identified inductively, to best represent the experiences of students, then grouped into thematic categories. Themes were named and refined through iterative analysis, returning to the transcripts to ensure they reflected the original data. Themes were validated through regular discussions between all researchers, ensuring reflexivity. Analysis focused on participant’s lived experiences (of symptoms, help-seeking and supporting others) as opposed to hypothetical help seeking scenarios, therefore, the accounts of those with personal experience were prioritized.

## Results

Participant characteristics are detailed in Table 1. All students described feeling “stressed” at times during their studies. Nine students had thought their symptoms might have been secondary to a mental health problem. Students interpreted the following signs as indicative of a possible mental health problem (with some students reporting multiple symptoms); suicidal thoughts (*n* = 3), social withdrawal (*n* = 5), non-attendance at the medical course (*n* = 3), depressed mood and/or anhedonia (*n* = 4), anxiety (*n* = 6) and PTSD symptoms (*n* = 1). Seven students sought help from healthcare services (Table 2), two of whom temporarily interrupted their studies. Eleven students described supporting other students experiencing mental health problems. Students described similar influences on their help-seeking decisions, whether from healthcare professionals or university pastoral services, with some nuances as described below. Therefore these are considered together.

**Table 1:** Student Characteristics

Student Characteristics	Number of Students
Gender	
Female	9
Male	5
Mature Student (completed degree before medicine)	2
Location of Schooling	
United Kingdom (UK)	11
International	3
Openness to perusing a psychiatric career	
Open to a possible career in psychiatry	8
Would not consider a psychiatric career	6

**Table 2:** Students’ previous use of services for mental health problems. Some students sought help from multiple sources

**Use of Healthcare Services**	**7**
Primary Care Physician	6
Psychiatrist / Secondary Care Mental Health Services	1
Private talking therapy	2
University talking therapy (psychotherapy / counselling / cognitive behavioural therapy)	6
Talking therapy in a group setting	1
**Use of University Services**	**8**
Tutors, Teaching and Clinical Staff (without a formal pastoral role)	3
Pastoral support staff including student advice service	6
Disabled Student Service	2
Mitigating / Extenuating Circumstances	4
Occupational Health	1
**Total Number of Students**	**14**

Discussion of help-seeking decisions centered around five themes: Theme 1 explored the meaning students attached to their symptoms; Theme 2 considered the meaning students attached to experiencing a mental health problem; Theme 3 related to students’ evaluation of the appropriateness of help-seeking; Theme 4 centered on students’ concern that help-seeking represented a loss of control; and Theme 5 described practical barriers. Findings from each of these themes is described below, with illustrative quotes.

Students’ perceptions of symptoms and the need for help were continually revisited and revised, being influenced by the nature of symptoms and external experiences. For instance, experiences of disclosure to social contacts or professionals, subsequently informed students’ formulations of their own symptoms and need for help.

“I never did anything to myself, but I was having thoughts… I was like, ‘okay, I think this is serious now’… [But] I remember being bounced back… with the doctor saying… ‘come back in two weeks if you still feel like this’… [so] I was like, okay… they think I’m fine… so it must be alright” Student 4

### Theme 1: The Meaning of Symptoms

Although most of the students perceived themselves as having a reasonable understanding of common mental health problems, this did not appear to increase recognition of symptoms in themselves.

“I was… really low [in] mood… I knew about depression and stuff… but somehow the penny didn’t drop… I didn’t actually accept that, like, deep down there was a problem.” Student 4

Many students conceptualized symptoms as reflective of “stress”, rather than possibly being a sign of a health problem. Furthermore, stress was perceived to be an unavoidable part of medicine and so normalizing symptoms allowed students to discuss stressors, whilst deflecting any discussion about the state of their mental health.

“As medical students… we’re so acclimatized to stress and… feeling low and having tough times because med school is like that… you just start thinking this is normal and you lose sight of when this is no longer normal and it’s become an actual issue, and we need to actually deal with them” Student 8

Some students who had noticed symptoms in others, described feeling uncomfortable bringing this up with them.

“It is a difficult topic to broach, even though I know her really well… putting mental health into words is difficult for both parties... It is seen as a weakness and most people don’t want that to be broadcast” Student 2

Feelings of embarrassment about mental health symptoms, meant that potentially useful feedback was not sought from peers to guide symptom interpretation.

“[I experience] embarrassment being vulnerable…one of the most intimate things you can do is like talk about how you’re feeling or coping or if you’re struggling, especially if things were really hard for you.” Student 11

As a result of their training, many students considered themselves to have a good understanding of strategies to enhance wellbeing including self care. The perception of symptoms as non-pathological and manageable through such approaches, removed the need for help-seeking.

“We are medical students, we know we need water, and we know about sleep hygiene and things like that. We don’t need to be told [by a doctor]” Student 2

### Theme 2: The Meaning and Implications of Illness

Many students perceived that services were purely for people who were “ill”, as opposed to “stressed”. A few explained how conceptualizing symptoms as being reflective of an illness felt like a pivotal moment in their help-seeking journey. Yet, this also carried risks of being given a formal diagnosis and self-stigma.

“You feel like [wellbeing services] are not for you… I hadn’t been told I had depression… [and] those services are for people who have had depression for like long periods of time… It’s like that you don’t want the diagnosis… you think that, you know, you're just going through a bit of a rough patch and… it’ll be fine.” Student 14

“I thought I was just a bit weird… I had a lot of stigma I was putting on myself” Student 4

For some students, although stress was considered to be an inevitable consequence of pursuing a medical career, being unable to cope represented a personal failing. Mental illness was considered to be a “weakness” not to be experienced by doctors, for whom there was an expectation to be “strong”.

“Medical school is tough… you really have to be strong enough… There’s this idea out there that… admitting that you have these [mental health] problems makes you weak, and you need to man up and get over it” Student 8

“People talk about depression… [They say] ‘it’s just a lack of coping, et cetera’… Fault doesn’t … come into any aspect of medicine. But you’re mentioning mental health… people start trying to… [assign] blame.” Student 7

The experience of mental health problems did not fit with the perceived norms associated with the medical profession, and several students expressed concerns about the impact of these problems on their future job prospects.

“The major barrier [to help seeking] is being afraid they will get struck off and they will never be allowed to be a doctor, because [they] are slightly depressed.” Student 12

Furthermore, for many students, studying medicine appeared to mean more than simply undertaking a degree. Much value was placed on being a medical student and for some, their self-worth had become enmeshed with their identity as a future doctor. For these students, the experience of mental health problems, and the associated sense of failure therefore threatened their self-identity and carried broader repercussions.

“We are all taught to be very, very proud of being medics… when you say "I am a medical student"… [it means] ‘I am quite clever and I obviously work quite hard to be doing this’… in a very kind of superior way” Student 1

“This mentality’s very strong I think in medical students… you work hard, you knuckle down, you push through and regardless of what’s going on, you can do well and succeed…And the idea of having to stop, or take a break, It’s awful” Student 11

These negative perceptions around the meaning of illness for medical students, in the context of broader societal stigma, engendered feelings of embarrassment and discouraged the disclosure of illness, even with friends. This reinforced feelings of difference and limited options for support.

“People who did need [support] and did want it would feel embarrassed about… needing it… then they couldn’t speak out about it wanting it.” Student 2

“I didn’t tell anyone at uni… there is an aspect of, "Why am I having a problem with this and no one else is?" And I didn’t necessarily want people to know, especially other medical students... I just don’t think they would have really understood… they'd think… I'm just making a fuss about nothing” Student 10

Therefore, the negative connotations students commonly attached to mental illness in the medical profession were not often challenged.

“within the medical school… [mental illness] is seen as a weakness and slightly less understood because [it’s] slightly less shared.” Student 2

### Theme 3: The Appropriateness of Seeking Help

Students considered whether seeking help was an appropriate response to their symptoms and this was partially dependent on their perception of whether a problem was ‘bad enough’ to warrant help. Yet, this was not a foregone conclusion, even amongst those who recognized they may be unwell.

“when I was struggling a bit, the thing that stopped me going to see anyone was… I didn’t feel like I had *severe* depression, or like *severe* anxiety. So I didn’t think going to see anyone would be like the thing to do… You kind of feel like the services are put on for people who are like really struggling to the point of like it’s severely affecting their life… It’s hard to know whether those services are for you or not.” Student 14

The further development of symptoms, and associated functional impairment, were viewed as indicative of a need for help, though some described experiencing significant functional impairment before contemplating accessing support.

“it came to a point where like, my room was a pigsty… my friends would… bring me food and the plates were there… covered in fungus, it was gross, there were maggots in my room at one point - it was bad.” Student 9

Some students questioned whether mental health problems constituted a valid reason to seek medical help. Such views were reinforced by students witnessing senior doctors being dismissive of mental health problems and minimizing the impact of these problems on patients.

“I have heard some consultants… just brushing off [a patient’s mental health] problems as if they didn’t exist or… weren't valid. And if … you see this, very senior doctors are thinking that certain conditions are unimportant or are things that you should just get on with yourself, and shouldn’t have to seek help about, then that kind of propagates that persona of having to be self-reliant” Student 12

Additionally, the students’ identity as future medical professionals led some to question the appropriateness of seeking help for their own problems, due to a perception that doctors should always remain care-givers and should not be seeking help.

“being a medical student or a doctor, you are supposed to be the one that helps people, you are not supposed to be the one that seeks help yourself. And I think that even as a medical student.” Student 12

Students’ medical background led them to believe that they should know better than to seek help inappropriately. Fear of making such an error discouraged help-seeking.

“I feel like if I tell someone that… I’m struggling with something and they go, ‘oh, so that’s not really that serious’, I dread that… I would rather not tell anyone than have to listen to that. “ Student 8

In addition to considering the overall appropriateness of seeking help, students also considered which setting and service was most appropriate to seek help from. Students seeking treatment tended to first consult their GP practice, whilst those seeking academic support consulted the student advice and wellbeing service, which also provided university support, e.g. applying for mitigating circumstances. The few students who disclosed their symptoms to staff during clinical clerkships only did so when the clinical environment was directly relevant to their mental health symptoms.

“I told my supervisor there about [my abortion]… that first week of obs and gynae... I just couldn’t even look at people, which was so different… I was just feeling really terrible.” Student 1

### Theme 4: Losing Control

Students commonly expected help-seeking to result in a diagnosis being made. Many felt that once made, this would carry stigma, which could potentially lead to unwanted consequences that they would have no control over.

“I didn’t want it to become a sort of diagnosis as such… I just wanted to get on and be like everyone else.” Student 10

“I still think there’s quite a stigma about having a diagnosis that’s a mental health condition.” Student 7

“I remember… looking at this leaflet and it being like, "If these symptoms are [present] this is a sign you might be depressed." And I thought, "Quite a lot of that fits." But I didn’t really want to go down that route… I wanted to try and brush this under the rug... I didn’t want it to take control.” Student 10

In light of preexisting ideas about the career implications of mental illness, a formal record of diagnosis triggered concerns about career consequences.

“When I went to my GP for SSRI’s, my first question was, “is this going to affect my career?” Student 8

As a result, concerns about confidentiality, particularly at a university level, posed a substantial barrier to seeking help. Given their understanding of doctors’ duty of confidentiality, most students perceived healthcare services to be more confidential than those provided by the university.

“[With] the doctor-patient relationship… things will remain confidential, unless there is a real, significant problem… With the university, the line is not quite as clear… they are not bound by the same rules as doctors… therefore their kind of test for breaking confidentiality is not quite as stringent”. Student 12

Yet many students still worried about being fully open about their state of mind with healthcare professionals. A few expressed uncertainties about what constituted “a risk of harm to yourself or to others” (Student 8) and feared their symptoms could be disclosed to the medical school. Amongst those who sought help, students were sensitive to the responses of clinicians during their consultations. When perceiving a threat to their confidentiality, albeit conveyed through non-verbal cues, they became more cautious about what they disclosed, leading to under-reporting of suicidal thoughts amongst those who experienced them.

“She just went, “hold on, did you just say you’d be better off dead? I’ll just take my notes out again and write this down”, and I went, oh god, I’m not entirely sure where this is going to go… if it could come back to bite me later… It stopped me talking then and there” Student 8

For some students, these concerns about confidentiality led them to avoid university counselling in favor of alternative services.

“I’d be more inclined to go to like [local charity which provides counselling] or something like that, that’s completely separate and you don’t have to sort of worry about any information being passed on.” Student 1

However, a few students had reservations about such alternative services because they would not understand the specific needs or experiences of medical students and would not be able to provide targeted support.

“I could be feeling overwhelmed by something and I could call the Samaritans, but they wouldn’t know… how medical school works or what the impact of certain things is… [I would like] someone who would know… the things we have to go through and the things we have to do, and so they’d be able to provide meaningful support.” Student 8

### Theme 5: Practical Barriers

Practical barriers to help seeking were less important to students than the concerns described above. However, seeking help involved a need to prioritize this over other activities. Therefore, full timetables and clerkships outside of the university city, generated tangible barriers to accessing services. These barriers were compounded by stigma and confidentiality concerns, as students avoided requesting time off to attend appointments as they did not want to disclose their problems to clerkship staff.

“Getting an appointment’s really difficult, especially when you’re on placement… you have to take a whole day out.” Student 6

“I had my own private counselling for a while last year. But… I’m not placed in [University City] now, it’s difficult to coordinate that. So I’ve stopped that… The downside of [distant clerkships] is… you can’t always plan things… [Also,] I couldn’t go and tell [clerkship staff about] my mental health issues… it’s not something I go on telling people” Student 8

## Discussion

Our qualitative study furthers understanding about the factors influencing medical students’ decisions to seek help for distress. Importantly, we have shown that some aspects of medical culture, and the tethering of self-worth to professional identity, may inhibit help-seeking among students. It extends the current literature by emphasizing the relevance of sociological perspectives on help-seeking and potentially offers insights for targeting novel approaches to supporting help-seeking behavior.

Students’ narratives reflected a complex interplay of individual, social, and cultural factors, which influenced how students perceived mental health symptoms and the potential need for support. For many, the perception of stress as being a normal feature of working as a doctor, as well as their confidence in self-care strategies, encouraged normalization of symptoms. This diminished the perceived need for professional support. Students’ professional identity as needing to be a “strong” caregiver (versus care-receiver), and professional norms which stigmatized mental illness, further discouraged help-seeking. Students’ social role as a medical student conflicted with the sick role. Such conflicting social roles have been postulated to influence meaning ascribed to symptoms and the prioritization of help seeking [29], which is supported by our findings.

Concerns around confidentiality and the perceived career implications of help-seeking were further compounded by the value students attributed to becoming a doctor, with this already forming an important part of their identity. Such concerns inhibited disclosure to university services. They also impaired the reporting of symptoms which the students believed might lead to their clinician breaking confidentiality in a healthcare setting, particularly suicidal thoughts. This finding underscores the need for direct inquiry about suicidality among distressed students, given their potential reluctance to volunteer such information. These factors, in addition to practical barriers, such as difficulty accessing services during clerkships, may explain the reluctance of medical students to seek help for mental disorder, despite possessing good mental health literacy.

The findings resonate with existing sociological and psychological models of help-seeking behavior. Students’ concerns that formal diagnosis represented an irreversible step which they would prefer to avoid due to career implications, echoes previous work which has raised perceived lack of confidentiality as inhibiting help-seeking [30]. Students' decisions were not only based on weighing benefits (chiefly, the resolution of distress and academic support) and costs (e.g. career implications), but aligned with sociological understandings of illness behavior. Specifically, help-seeking comprised a series of iterative decisions based on students’ interpretation of their experience, as opposed to the experience itself, and was informed by the social context in which decisions arise [18, 19, 31]. For example, students interpreted symptoms through the lens of medical culture as unavoidable "stress", and their judgments about the appropriateness of help-seeking were informed by the perceived attitudes of senior doctors whose clinical practice they observed. Concerns about diagnosis and help-seeking appeared to reinforce non-pathological interpretations of symptoms, with students wanting to “brush them under a rug” (Student 10). This finding resonates with the Cycle of Avoidance Model [21] which describes how individuals employ strategies like normalization to avoid negative consequences associated with formal help-seeking. These findings extend such conceptualizations by identifying the key role of identity. Klik’s work on Social Identity Perspective demonstrates how self-identity as someone with mental illness drives help-seeking [20]. Our study has demonstrated how the ‘positive in-group distinctiveness’ [32] perceived by those with a self-identity as a medical student, may inhibit help-seeking, as students derived self-worth from this identity, saw mental illness as incompatible with the expected "strength" of doctors, and feared career implications of mental health problems, potentially jeopardizing their membership of this professional group. These findings emphasize the need for medical educators to recognize the risks of reinforcing the superiority of the medical identity, over students’ personal identities outside of medicine. Medical schools should actively challenge stigma and professional norms that equate help-seeking with weakness, fostering an environment where faculty role-modelling of healthy behaviors is encouraged. Inclusion of interventions in which doctors share personal experience of illness and recovery within curricula, may improve attitudes to help-seeking [33].

The study has both strengths and limitations. The qualitative design provided rich, in-depth insights but transferability was limited by a small sample size (*N* = 14) and single-institution setting. As a result, it is possible that cultural factors specific to the UK and/or the institution may have influenced students' help-seeking behavior. However there is evidence of unmet need for mental health support amongst medical student populations globally, highlighting this as an international issue [2]. The low initial response rate raises the possibility of response bias—students with a disproportionately high prevalence of mental health problems and help-seeking behavior—may have been attracted to participating in the study. The recruited sample may also have differed from the general medical student population on other characteristics (e.g. ethnicity), not captured during data collection. Purposive sampling ensured a diversity of students including those with symptoms who had and who had not sought help. However, the lack of an objective measure of symptom severity prevented assessment of the clinical appropriateness of participants' decisions regarding help-seeking, and it is likely that some of those who did not seek help did not need it. Nonetheless all students experienced stress, nine had thought their symptoms may be due to a mental health problem and seven sought help (though sometimes after a protracted period), therefore we elicited varied accounts of help-seeking decisions from students who had faced this decision themselves.

The individual interviews and longitudinal qualitative methodology enabled the interviewer to build a good rapport with students, facilitating detailed discussion of a sensitive topic. Equally, the fact that the interviewer is a psychiatrist may have influenced students’ disclosure, as well as data interpretation, given her clinical knowledge and experience of medical school. Because of these concerns, a second perspective was also garnered through independent second coding of a sample of transcripts by co-author LB (a social scientist).

In conclusion, the findings suggest some potential strategies to promote help-seeking among medical students. Confidential services accessible to busy students during clerkships, which validate medical students’ help-seeking behavior, may enhance students’ ability to access support. Clinicians supporting students should be aware of potential under-reporting of suicidal thoughts, suggesting a need to use direct probing questions. However, since much of the process of reaching a help-seeking decision occurs before students encounter services, systemic approaches are required to address destructive stigma towards mental illness (including among senior medics); to challenge perceived norms which discourage help seeking; and to address the negative perceptions among medical students towards the efficacy of psychiatric treatments. Education, both of students and the multidisciplinary medical professionals delivering their curriculum, will help to address these systemic issues, with the medical curriculum providing a key platform to shape student attitudes towards mental health problems. Role-modelling healthy behaviors and attitudes towards mental health should be a responsibility of all doctors. Prioritizing psychiatry teaching and clinical experience within curricula is a clear way that medical schools can highlight the importance of mental health, with direct patient contact having beneficial effects on student attitudes [26, 34, 35]. Reframing boundary-setting and utilizing support not as personal failings, but as professional competencies, may challenge the admiration of short-term high pressured working over sustainable practice across a long career, shifting students’ definition of a successful doctor. Supporting students to engage in self-care behaviors is important, however wellbeing initiatives must also emphasize the limitations of self-care, and encourage students to recognize when they need professional help. Future research should investigate the prevalence and impact of the barriers to help-seeking among medical students, across diverse institutional contexts. Interventions are needed to mitigate the impact of these barriers and these interventions should be subjected to rigorous evaluation.

## Data Availability

Anonymised quotes from the dataset supporting the conclusions of this article are included within the body of the article. The full transcripts generated and analysed during the current study are not publicly available due to the risk this would pose to the anonymity of the individual participants. Excerpts of the transcripts relevant to the study are available from the corresponding author on reasonable request.
